# Exploring diabetes care at the Leicester Diabetes Centre

**DOI:** 10.51866/mol.921

**Published:** 2025-05-05

**Authors:** Shafinaz Syed Hamzah Shariffah

**Affiliations:** 1 MBBChBAO, FRACGP, Klinik Kesihatan Simpang Kuala, Alor Setar, Kedah, Malaysia. Email: finazhamzah@gmail.com

**Keywords:** Diabetes Mellitus/education, Glucose Monitoring, Continuous, Patient Education as Topic, Diabetes Mellitus, Type 1, Diabetes Mellitus/therapy

Our journey into diabetes care took an exciting turn when my colleagues, Dr Mahani Kamaruddin and Dr Salinah Mohd Mudri, and I embarked on a clinical observership at the Leicester Diabetes Centre (LDC). The LDC is recognised globally for diabetes research and training and collaborates with the University of Leicester and the University Hospitals of Leicester NHS Trust.

After months of planning, we were granted a 1-week clinical observership in January 2025. We arrived in Leicester on a cold and bleak evening, welcomed by temperatures ranging from 6°C to 9°C. Leicester, a city rich in history and diversity, set the stage for our learning experience.

On our first day, Ms Hannah Wakefield, our programme coordinator, warmly welcomed us. The day began with an orientation, followed by an introduction to the LDC’s multidisciplinary team (MDT). At the diabetes outpatient clinic, we observed a diabetes specialist nurse, Grace Grudgings, during an MDT clinic session. Continuous glucose monitoring (CGM) devices were available for patients, and digital networking between hospitals, pharmacies and general practitioners facilitated seamless diabetes care. The structured appointment system ensured minimal waiting times and an efficient workflow.

**Figure 1:**
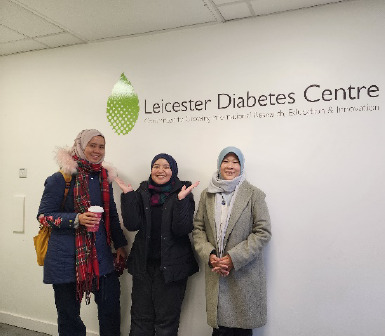
At Leicester Diabetes Centre. From right to left: Dr Salinah, Dr Sharifffah Shafinaz and Dr Mahani.

**Figure 2:**
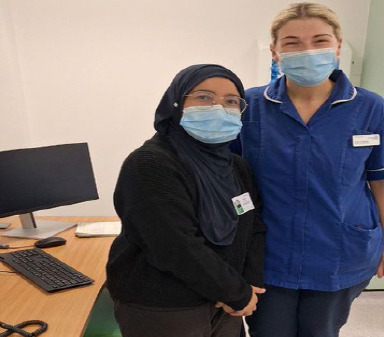
A session with a diabetes specialist nurse, Grace Grudgings, at the diabetes outpatient clinic of the Leicester Diabetes Centre.

**Figure 3:**
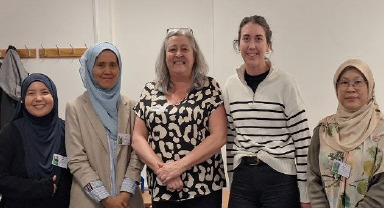
Diabetes Education and Self-Management for Ongoing and Newly Diagnosed (DESMOND) programme session at the Leicester Diabetes Centre with DESMOND trainers, Denise Orwin and Hannah Walters.

On the next day, we observed a training session for the renowned Diabetes Education and SelfManagement for Ongoing and Newly Diagnosed programme. This internationally recognised initiative empowers patients and caregivers by enhancing their knowledge and self-management skills. Although we could not take photographs due to copyright restrictions, we gained valuable insights into structured diabetes education.

Midweek, we participated in the Type 1 Diabetes Experience Day, part of the Eden Healthcare Professional Training programme. We met Mr James Ridgeway, a diabetes specialist nurse with type 1 diabetes (T1D). Interactive sessions covered decision-making in T1D management, dietary considerations and psychological well-being. We also learnt about public health initiatives in the United Kingdom, such as the ELSA and T1DRA screening programmes, which identify individuals at risk forTID and facilitate early interventions including teplizumab therapy to delay disease onset.

**Figure 4:**
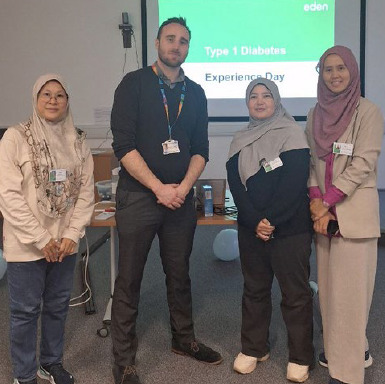
Type 1 Diabetes Experience Day session with diabetes specialist nurse, James Ridgeway.

On the fourth day, we engaged in a CGM webinar led by Mr Ridgeway. The session focused on interpreting ambulatory glucose profiles using FreeStyle Libre and Dexcom CGM, deepening our understanding of how these devices enhance diabetes management.

Our final day in Leicester brought another highlight - observing a diabetes outpatient clinic at Glenfield Hospital with consultant endocrinologist Dr Ann Morrison. She shared insights into novel diabetes therapies, including Tirzepatide, a once-weekly injectable recommended for patients with type 2 diabetes with a body mass index over 30 kg/m^2^. Patients on Tirzepatide reported improved glycaemic control, reduced variability and significant weight loss, making it a promising treatment option.

**Figure 5:**
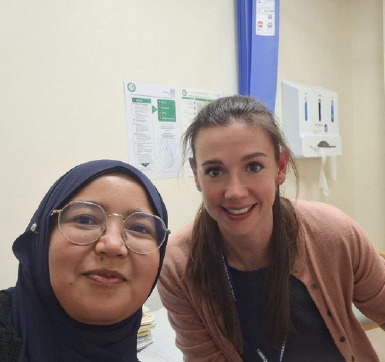
A session with Dr Ann Morrison, a consultant endocrinologist, at the diabetes outpatient clinic of Glenfield Hospital.

Reflecting on our time at the LDC, we were inspired by their structured approach to diabetes care, the integration of technology and the emphasis on patient education. This experience reinforced our commitment to adopting similar strategies in Malaysia, ensuring patients receive the best diabetes care through innovation and education.

